# A Remodelação do Sistema Nervoso Autônomo Cardíaco pode Desempenhar um Papel na Fibrilação Atrial: Um Estudo do Sistema Nervoso Autônomo e Receptores Miocárdicos

**DOI:** 10.36660/abc.20200725

**Published:** 2021-08-04

**Authors:** Ítalo Martins de Oliveira, Evilásio Leobino da Silva, Yasmin de Oliveira Martins, Hermano Alexandre Lima Rocha, Maurício Ibrahim Scanavacca, Paulo Sampaio Gutierrez

**Affiliations:** 1 Hospital das Clínicas Faculdade de Medicina Universidade de São Paulo São Paulo SP Brasil Instituto do Coração (InCor), Hospital das Clínicas da Faculdade de Medicina da Universidade de São Paulo , São Paulo , SP - Brasil; 2 Hospital Messejana de Coração e Pulmão Dr. Carlos Alberto Studart Gomes Fortaleza CE Brasil Hospital Messejana de Coração e Pulmão Dr. Carlos Alberto Studart Gomes , Fortaleza , CE - Brasil; 3 Hospital Geral de Fortaleza Fortaleza CE Brasil Hospital Geral de Fortaleza (HGF), Fortaleza , CE - Brasil; 4 Harvard T.H. Chan School of Public Health Boston EUA Harvard T.H. Chan School of Public Health , Boston - EUA

**Keywords:** Fibrilação Atrial/fisiopatologia, Sistema Nervoso Autônomo, Neurotransmissores, Miocárdio

## Abstract

**Fundamento:**

Alterações do substrato elétrico e anatômico do coração são fatores que originam e perpetuam a fibrilação atrial (FA), porém, os mecanismos envolvidos não foram totalmente elucidados ainda. Objetivo: Avaliar o papel do remodelamento do sistema nervoso cardíaco intrínseco (SNCI), incluindo fibras nervosas e receptores muscarínicos e β-adrenérgicos, na FA permanente humana.

**Métodos:**

Foram avaliadas 4 amostras em átrios de 13 corações obtidos em necrópsias de pacientes com doença cardíaca e FA permanente, e em 13 controles com as mesmas doenças, porém, sem FA. Utilizando imunoperoxidase e histomorfometria, quantificamos a densidade das fibras do SNCI, bem como a porcentagem positiva de miocárdio para receptores β-adrenérgicos 1, 2 e 3, receptor quinase 5 acoplado à proteína G (GRK-5), e receptores muscarínicos 1 a 5. Os resultados foram comparados usando ANOVA e ANOVA hierarquizada e ajustados pelo volume do átrio esquerdo e, para avaliação da expressão de receptores β e GRK-5, pelo uso de β-bloqueadores. Adotamos como significativo α = 0,05.

**Resultados:**

Houve aumento na densidade das fibras ( *p* <0,01), especialmente nas fibras simpáticas ( *p* =0,02). Quanto aos receptores muscarínicos, só houve diferença nos M1, que estavam aumentados (5,87±4,52 *vs* 2,85±2,40; *p* =0,03). Quanto aos componentes do sistema adrenérgicos analisados, houve expressão aumentada de β-3 (37,41 *vs* 34,18, *p* =0,04) e GRK-5 (51,16 *vs* 47,66; p<0,01). O uso de β-bloqueadores não teve impacto na expressão de receptores beta.

**Conclusão:**

O aumento na inervação do SNCI e a alteração na expressão de receptores em regiões suscetíveis de desencadear FA podem ter um papel na fibrilação atrial permanente.

## Introdução

Alterações nos substratos elétrico e anatômico do coração constituem o fator primário que origina e perpetua a fibrilação atrial (FA). Em pacientes com FA sem doença estrutural do coração, focos ectópicos originados nas veias pulmonares têm papel bem definido como desencadeante de FA paroxística. ^1^ No entanto, a FA é, na maior parte dos casos, secundária a doenças cardíacas estruturais, como doença isquêmica do coração, doenças valvares e outras, que apresentam consequências hemodinâmicas e anatômicas, tais como aumento do átrio esquerdo, que estão associadas à progressão da arritmia. ^1^

A fibrose é também amplamente vista como fator independente relacionado à FA persistente em corações com alterações estruturais. ^2^ Ela, porém, não explica totalmente a arritmia, sendo mais associada às doenças subjacentes do que à FA persistente em si. ^3^

A avaliação eletrofisiológica demonstrou não só uma efetiva heterogeneidade do período refratário, mas também a anisotropia das propriedades de condução, tanto nas veias pulmonares quanto nos seus óstios atriais, o que pode causar a reentrada de estímulos elétricos. ^4^ Entretanto, os mecanismos cruciais que governam a perpetuação da FA não foram elucidados por completo - o que se reflete em resultados modestos no tratamento de pacientes com FA persistente prolongada. ^5^

Estudos básicos e clínicos sugeriram haver participação significativa do sistema nervoso autônomo cardíaco intrínseco (SNACI) no desencadeamento e na manutenção da FA. ^6 , 7^ A ativação do SNACI pode causar mudanças importantes no período refratário atrial, inclusive aumento na dispersão da refratariedade, que é um dos importantes mecanismos de desenvolvimento da FA persistente. ^1 , 8 - 10^

Estudos experimentais mostraram hiperinervação simpática em cães com FA, e aumento na inervação simpática e parassimpática em áreas relacionadas a essa arritmia em animais com insuficiência cardíaca. ^11^ Uma relação entre o SNACI e a FA foi também relatada em seres humanos, porém, em comparação com pacientes saudáveis. ^2 , 12^ A possibilidade de envolvimento de distúrbios em fibras e receptores do SNACI na fibrilação atrial humana foi pouco explorada.

Assim, o objetivo do presente estudo foi avaliar o sistema nervoso autônomo cardíaco intrínseco, incluindo fibras simpáticas e parassimpáticas, e a expressão atrial de cinco tipos de receptores muscarínicos e três adrenérgicos, assim como do receptor quinase 5 acoplado à proteína G (a qual controla a expressão dos receptores adrenérgicos). Estudamos corações de pacientes com doença estrutural e fibrilação atrial permanente e, como controles (o que foi um fator importante), os de pacientes portadores das mesmas doenças, mas sem fibrilação atrial.

## Métodos

Este estudo foi guiado pelos princípios da Declaração de Helsinque e aprovado pela Comissão Científica do Instituto do Coração (InCor) do Hospital das Clínicas, da Faculdade de Medicina da Universidade de São Paulo, São Paulo, Brasil (#SDC 3043/07/118).

### Pacientes

Foram utilizadas partes das amostras de estudo anterior. ^3^ Analisamos 13 corações de adultos (acima dos 18 anos) com FA permanente registrada em prontuário com duração mínima de 2 anos, ^1^ que foram submetidos a necrópsia (realizada em até menos de 24 horas após a morte) no Laboratório de Anatomia Patológica do *InCor* . Todos os pacientes tinham doenças subjacentes: doença isquêmica do coração (4), valvopatias (4), miocardiopatia hipertensiva (2), miocardiopatia dilatada idiopática (2) ou doença de Chagas, forma crônica cardíaca (1). Para evitar fatores de interferência ligados às doenças de base, corações de 13 pacientes submetidos a necrópsia no mesmo laboratório foram selecionados como controles, com pareamento de acordo com as doenças dos que tinham fibrilação atrial, mas sem qualquer referência a essa arritmia em seus prontuários. Em ambos os grupos, pacientes que tivessem sido submetidos a cirurgia ou a qualquer procedimento com potencial de modificar a estrutura cardíaca ou com cardiopatias congênitas foram excluídos.

### Amostras dos corações

**Figura 1:**
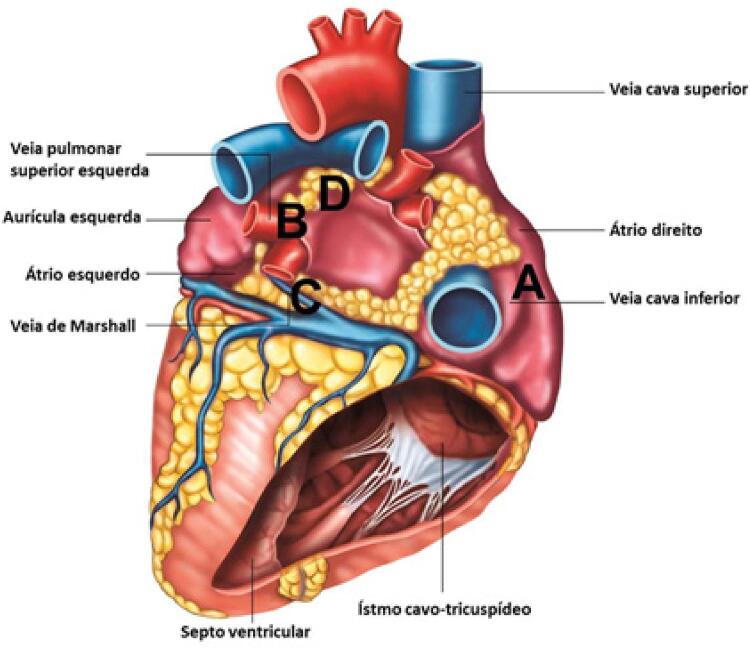
– *Imagem fotorrealística de vista posterior do coração humano. Quatro amostras foram coletadas nas seguintes localizações: A) parede posterior do átrio direito. B) junção da veia pulmonar superior esquerda com o átrio esquerdo; C) Segmento medial da rota da veia de Marshall; D) coxim gorduroso superior esquerdo.*

De cada coração foram obtidas quatro amostras, contendo epicárdio, miocárdio e endocárdio: na parede posterior do átrio direito ( Figura 1A ); na junção da veia pulmonar superior esquerda com o átrio esquerdo ( Figura 1B ); na porção medial do trajeto da veia de Marshall ( Figura 1C ); e em torno do coxim gorduroso superior esquerdo (os coxins gordurosos são concentrações de gordura epicárdica nas quais há tecido nervoso, sendo conhecidos como *fat-pads* ). Tais áreas foram escolhidas porque foram antes implicadas na fibrilação atrial, e são comumente analisadas em outros estudos, ^3 , 12 , 13^ com exceção da parede posterior do átrio direito, selecionada para verificar se eventuais alterações seriam difusas nos átrios. As localizações são apresentadas na figura 1 .

Após processamento histológico convencional e embebição em parafina, cortes dessas amostras com 4 micrômetros de espessura foram preparados para a quantificação da inervação autonômica, receptores adrenérgicos e muscarínicos e expressão de GRK-5.

### Quantificação dos receptores e das fibras nervosas autonômicas

A positividade forte para receptores adrenérgicos e muscarínicos, GRK-5 e área total de miocárdio considerada foram medidas por detecção automática de cor em 3 campos microscópicos em cada lâmina. Para evitar viés de seleção na escolha dos campos, foram analisados os mais distantes da etiqueta das lâminas.

As fibras nervosas autonômicas também foram quantificadas nas amostras. A proteína S-100 tem positividade em todos os nervos, enquanto a tirosina hidroxilase (TH) marca apenas as fibras adrenérgicas (simpáticas) pós-ganglionares. Assim como outros autores, ^14^ avaliamos custo-efetividade e consideramos os nervos TH-positivos como pertencendo ao sistema nervoso simpático; já os parassimpáticos corresponderam aos positivos para S-100 e negativos para TH. Diferentemente do que o empregado com os receptores, os cortes histológicos foram analisados por inteiro, e suas áreas e números de nervos foram quantificados em cada lâmina. Foram, então, calculadas as seguintes variáveis: porcentagem de área positiva (área positiva/ área do corte), densidade média de nervos positivos (número de nevos positivos/ área do corte) e área média dos nervos (área positiva/ número de nervos). Calculamos também o número total de fibras nervosas (S-100 positivas), fibras nervosas simpáticas (TH-positivas) e fibras nervosas parassimpáticas (S100-positivas e TH-negativas, diferença entre o total de fibras e as simpáticas).

Para aumentar o contraste entre positividade fraca e forte, as diluições para os receptores e o GRK-5 foram supraótimas ^15^ quando comparadas ao padronizado em outros tecidos. Como controle das reações, o anticorpo primário foi omitido em 5 lâminas escolhidas ao acaso. Os cortes foram examinados em sistema de análise de imagens *Axiovision 4.6,* acoplado ao microscópio *Axion imager A1* (ambos da *Carl Zeiss* , Alemanha) por observador que desconhecia a que grupo pertenciam as lâminas.

Especificação e diluição dos anticorpos: receptor muscarínico 1 (AB5164)- 1:100; receptor muscarínico 2 (AB9452)- 1:800; receptor muscarínico 3 (AB9451)- 1:200; receptor muscarínico 4 (AB9219)- 1:400; receptor muscarínico 5 (AB9453)- 1:400; receptor adrenérgico β1 (SC568) - 1:200; receptor adrenérgico β2 (SC570) - 1:50; receptor adrenérgico β3 (SC1473) - 1:20; quinase GRK-5 (SC 565) - 1:200; S-100 (Z0311) - 1:300; tirosina hidroxilase (MAB318) - 1:50.

O anticorpo para S-100 foi fornecido por *Dako* , Dinamarca; os anticorpos para tirosina hidroxilase e receptores muscarínicos, por *Chemicon* , Estados Unidos da América; e os anticorpos para receptores adrenérgicos e GRK-5, por *Santa Cruz Biotechnology* , Estados Unidos da América.

### Análises estatísticas

Inicialmente, as frequências absolutas e relativas foram calculadas para as variáveis categóricas, e as medidas de tendência central e dispersão para as numéricas. Para comparar casos com controles foram utilizados os testes de qui-quadrado e t de Student. Os testes paramétricos foram usados após o teste de Kolmogorov-Smirnov para avaliação da normalidade em todas as variáveis, e estimativas de erros robustos foram usadas em modelos regressivos. A análise de variância (ANOVA) *one-way* foi aplicada considerando-se cada conjunto de amostras iguais para identificar as diferenças entre elas. A análise de covariância foi também realizada para ajuste das dimensões do átrio e uso de β-bloqueadores, quando apropriado, ao analisar os cortes individuais. Modelos lineares gerais (também conhecidos como análise de variância hierarquizada [ *nested* ANOVA]) de todas as amostras histológicas dos participantes individuais) foram também aplicados para identificar o impacto do determinante principal (a saber, tratamento, um fator intersujeitos) nas diferentes variáveis dependentes. Finalmente, múltiplos modelos hierarquizados lineares gerais foram aplicados a todos os cortes histológicos para cada caso. Consideramos significantes valores de p iguais a ou menores que 0,05. Em todos os modelos, foram realizados ajustes de Bonferroni nos valores de p. As análises foram efetuadas no programa SPSS, versão 23 ( *IBM, Inc* , Estados Unidos da América).

Assim como em nosso estudo prévio de aspectos histológicos, inclusive fibrose, já que o volume atrial difere entre pacientes com e sem fibrilação atrial, fizemos a análise de sensibilidade com métodos de ajuste considerando as diferenças no tamanho do átrio esquerdo. A seguir, estimamos os resultados de cada variável em corações de qualquer grupo com um tamanho específico de átrio esquerdo para verificar se potenciais diferenças entre grupos poderiam estar ligadas a essa covariável. Adicionalmente, o uso de β-bloqueadores foi incluído para ajuste da avaliação de receptores β-adrenérgicos e de GRK-5.

## Resultados

As características clínicas, morfológicas e ecocardiográficas dos pacientes com fibrilação atrial permanente e seus controles são mostradas na tabela 1 .

**Tabela 1:** – Dados clínicos e ecocardiográficos de pacientes com FA permanente e controles

Variáveis	Casos com FAp (n=13)	Casos com FAp (n=13)	p
Pacientes do sexo masculino [n/(%)]	5 (38,5)	8 (61,5)	0,24
Idade (anos) [média/(dp)]	67,5 (15,4)	65,5 (11,4)	0,71
**Doença cardíaca subjacente [n/(%)]**			
Doença isquêmica do coração	4 (30,8)	4 (30,8)	
Doença da válvula, incluindo DR	4 (30,8)	4 (30,8)	
Cardiopatia hipertensiva	2 (15,4)	2 (15,4)	
Cardiomiopatia dilatada idiopática	2 (15,4)	2 (15,4)	
Doença de Chagas	1 (7,7)	1 (7,7)	
Peso (kg) [média/(dp)]	66,5 (14,1)	63,8 (15,0)	0,67
Altura (cm) [média/(dp)]	162,4 (14,7)	160,8 (8,8)	0,78
IMC (kg/m ^2^ ) [média/(dp)]	25,0 (2,9)	24,5 (4,2)	0,74
Diabetes *mellitus* [n/(%)]*	3 (23,1)	3 (25,0) (n=12)	0,99
Uso de beta-bloqueadores	5 (38,4)	5 (38,4)	
Hipertensão arterial sistêmica - [n/(%)]*	9 (69,2)	4 (33,3) (n=12)	0,07
Volume de átrio esquerdo no eco [média/(dp]	83,2 (38,4)	47,9 (40,8)	0,03
Espessura do septo do VE [média/(dp)]	10,3 (2,4)	10,4 (1,6)	0,94
Fração de ejeção do VE [média/(dp)]	49,8 (20,1)	46,1 (19,8)	0,67
Razão colágeno/colágeno+miocárdio [média +(dp)]	0,26 (0,09)	0,23 (0,06)	0,35

FAp: fibrilação atrial permanente; n: número de casos; dp: desvio padrão; DR: doença reumática; IMC: índice de massa corporal; * sem informação sobre um paciente controle; eco - ecocardiograma; VE: ventrículo esquerdo. Adaptado de Oliveira IM et al.3

**Figura 2:**
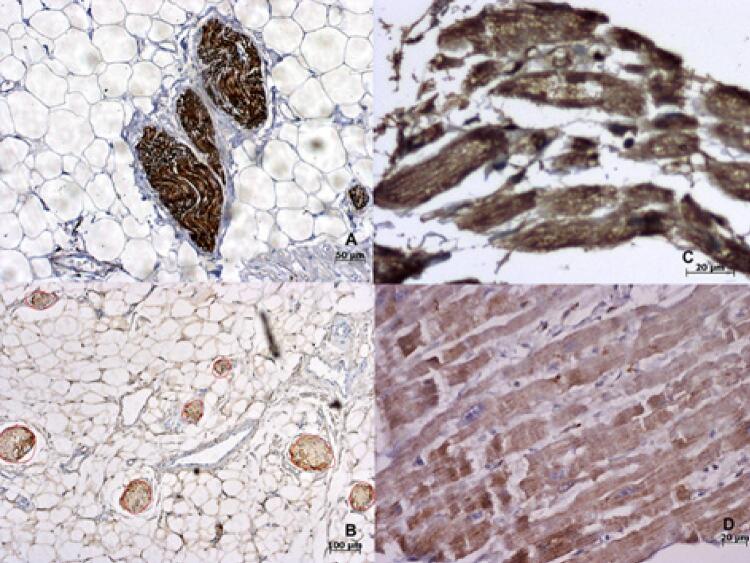
– *A) Fibras nervosas fortemente positivas para tirosina-hidroxilase, portanto consideradas fibras simpáticas; B) Fotomicrografia da tela do sistema de análise de imagens mostrando nervos marcados para proteína S-100; C e D) Áreas positivas (C) e negativas (D) em cortes histológicos de miocárdio com reação imuno-histoquímica para o receptor muscarínico 1.*

Dados relativos às fibras nervosas, considerando cada território amostrado, bem como todos em conjunto, são apresentados na tabela 2 . Levando-se em consideração separadamente cada localização, não são observadas diferenças quanto à densidade de fibras autonômicas intrínsecas. A análise englobando todas as amostras demonstra aumento de nervos simpáticos nos pacientes com FA (8,53±20,25/cm ^2^ *vs* 2,67±4,57/cm ^2^ , p=0,04). Após ajuste quanto ao tamanho do átrio esquerdo, aparece um aumento também nos nervos parassimpáticos e na quantidade total de nervos. A figura 2 (A e B) mostra a imunoexpressão das fibras nervosas em nossas amostras.

**Tabela 2:** – Fibras nervosas autônomas de corações de pacientes com FA permanente e de controles

Fibras	Todos (S100) (unidades/cm ^2^ )		Nervo simpático (TH+) (unidades/cm ^2^ )		Nervo parassimpático (TH-) (unidades/cm ^2^ )	
Grupo	FAp	Controle	FAp	Controle	FAp	Controle
AD - parede posterior	8,85±9,40	9,10±5,15	0,37±0,99	0,50±1,14	8,48±9,57	8,59±5,07
	p 0,935,	0,710 ^¥^	p 0,753,	0,905 ^¥^	p 0,971,	0,700 ^¥^
AE - junção da veia pulmonar superior esquerda	41,61±35,79	25,78±20,90	19,74±34,26	4,95±6,78	21,86±14,78	20,83±20,47
	p 0,181,	0,256 ^¥^	p 0,140,	0,158 ^¥^	p 0,884,	0,918 ^¥^
AE - meio da rota da veia de Marshall	40,15±60,28	14,90±9,48	5,58±9,56	2,39±4,76	34,56±58,07	12,51±9,48
	p 0,149,	0,390 ^¥^	p 0,292,	0,230 ^¥^	p 0,189,	0,500 ^¥^
FP - superior à esquerda	38,05±55,72	19,25±11,95	8,42±16,07	2,85±2,82	29,62±40,56	17,47±10,53
	p 0,246,	0,637 ^¥^	p 0,248,	0,666 ^¥^	p 0,325,	0,681 ^¥^
Amostras em conjunto	32,16±45,76	17,26±14,20	8,53±20,25	2,67±4,57	23,63±36,77	14,80±13,27
	p 0,136 ^e^ ,	0,001 ^Ϯ^	p 0,044 ^e,^	0,017 ^Ϯ^	p 0,237 ^e^ ,	0,001 ^Ϯ^

*Dados apresentados como média ± desvio padrão. FAp: fibrilação atrial permanente; AD: átrio direito; AE: átrio esquerdo; FP: coxim gorduroso (“fat pad”). p valor ANOVA não ajustado, ^¥^ ANOVA ajustado pelo tamanho de átrio esquerdo; ^e^ ANOVA hierarquizado não ajustado; ^e^ ANOVA hierarquizado ajustado pelo tamanho de átrio esquerdo.*

Os resultados da expressão de receptores muscarínicos e adrenérgicos e de GRK-5 são apresentados na tabela 3 . Estão divididos conforme a localização das amostras, havendo ainda dados da reunião de todas elas.

**Tabela 3:** – Expressão dos receptores muscarínicos e β-adrenérgicos em corações de pacientes com AF permanente e controles

Receptor	Grupo	AD - parede posterior			AE - ponto médio da veia Marshall			AE - junção da veia pulmonar superior esquerda			AE - perto da coxim de gordura superior esquerda			Amostras em conjunto		
			p	p*		p	p*		p	p*		p	p*		p	p**
M1	FAp	6,47±3,39	0,001	0,002	5,56±4,64	0,021	0,131	6,32±5,33	0,286	0,270	5,15±4,89	0,038	0,220	5,87±4,52	<0,001	0,032
	Controle	2,77±1,38			2,22±1,48			4,30±4,03			2,12±0,93			2,85±2,40		
M2	FAp	7,60±5,95	0,762	0,982	5,64±3,54	0,110	0,066	7,84±4,13	0,198	0,107	5,65±2,41	0,039	0,038	6,69±4,26	0,760	0,666
	Controle	6,93±5,15			3,73±2,12			14,24±16,88			3,62±2,41			7,14±9,73		
M3	FAp	43,50±19,08	0,105	0,315	37,61±20,97	0,296	0,281	41,90±18,88	0,546	0,281	31,00±13,27	0,025	0,151	38,51±18,34	0,069	0,291
	Controle	31,04±18,61			29,10±18,80			46,50±19,61			20,10±9,58			31,71±19,21		
M4	FAp	9,14±5,47	0,201	0,169	9,90±6,67	0,023	0,049	7,64±4,00	0,690	0,618	8,18±11,72	0,192	0,678	8,71±7,37	0,016	0,213
	Controle	5,76±4,74			4,44±4,56			8,42±5,66			3,76±1,95			5,59±5,45		
M5	FAp	18,94±11,93	0,302	0,704	12,90±11,34	0,368	0,645	20,92±22,81	0,737	0,946	12,06±9,32	0,570	0,977	16,21±14,88	0,212	0,507
	Controle	14,51±9,37			8,67±12,14			18:30±15,91			9,83±10,39			12,83±12,47		
β1	FAp	43,90±12,39	0,975	0,742	47,59±21,40	0,036	<0,001	37,48±21,90	0,438	0,288	40,23±22,37	0,552	0,214	42,05±19,75	0,295	0,520
	Controle	44,10±17,81			28,98±19,34			43,60±17,42			34,89±22,83			37,89±19,93		
β2	FAp	23,81±11,96	0,785	0,445	32,42±19,20	0,180	0,589	20,57±13,48	0,323	0,257	23,47±16,69	0,037	<0,001	24,80±15,61	0,408	0,081
	Controle	25,48±17,51			23,04±13,88			27,63±21,32			12:38±7,00			22,14±16,46		
β3	FAp	39,32±20,29	0,911	0,469	36,36±26,36	0,422	0,940	36,45±11,81	0,351	0,281	37,50±18,53	0,177	0,314	37,41±18,17	0,406	0,039
	Controle	38,40±20,45			29,04±20,40			42,38±19,10			26,89±20,31			34,18±20,53		
GRK5	FAp	49,81±18,49	0,899	0,976	44,84±18,78	0,999	0,147	53,43±15,28	0,796	0,862	55,45±16,53	0,086	0,320	51,16±17,17	0,284	<0,001
	Controle	50,53±7,61			44,85±19,75			52,95±13,26			43,29±18,00			47,66±15,39		

Dados apresentados como proporção média (%) ± desvio padrão. FAp: fibrilação atrial permanente; AD; átrio direito; AE:átrio esquerdo; GRK5: receptor acoplado à proteína G. p valor ANOVA não ajustado. *Anova ajustada pelo tamanho do átrio esquerdo para M1 a M5, e pelo volume de átrio esquerdo e uso de β-bloqueadores em β1 a β3 e GRK-5.*Anova hierarquizado ajustado pelo tamanho de átrio esquerdo para M1 a M5 e pelo tamanho de átrio esquerdo e uso de bloqueador de β em β1 a β3 e GRK-5.

A imunomarcação de receptores muscarínicos é mostrada na figura 2, C e D . Não existe diferença considerável entre as regiões subepicárdica e subendocárdica. Em corações de pacientes com FA permanente, a expressão de todos os tipos de receptores muscarínicos, com exceção do 5, estava aumentada em ao menos um território. Houve mais alterações no coxim gorduroso superior esquerdo e na veia oblíqua do átrio esquerdo (veia de Marshall). No entanto, após ajuste quanto ao tamanho do átrio esquerdo, apenas a expressão de M1 no átrio direito (e, consequentemente, a avaliação global) e M2 junto ao coxim gorduroso permaneceram significantes.

Em relação aos receptores β-adrenérgicos e GRK-5, não foi encontrada diferença na análise global dos subtipos 1 e 2 (apenas aumento em uma amostra de cada). Porém, β-3 e GRK-5 apresentaram aumento em todas as localizações na análise ajustada. Não foi detectada diferença entre pacientes que tomavam β-bloqueadores e os que não o faziam (dados não apresentados).

## Discussão

O sistema nervoso autônomo cardíaco intrínseco na fibrilação atrial permanente

O SNACI corresponde a uma rede neural composta por fibras nervosas e plexos ganglionares (simpáticos e parassimpáticos) encontrados no coração e nas grandes veias adjacentes. ^16^ Tem papel importante na fisiopatologia da FA, como demonstrado por estimulação elétrica ou por injeções de parassimpatomiméticos. ^17^ Os dados atuais indicam não somente uma função importante na ativação dos eixos simpático e parassimpático, mas também que a modificação do balanço entre suas ações está envolvida na iniciação da FA. ^8 , 18^

Neste estudo, realizamos análise abrangente do SNACI, enfocando tanto nos nervos quanto nos receptores muscarínicos e beta-adrenérgicos. Observamos aumento de fibras nervosas autonômicas atriais, em particular dos nervos simpáticos. Entretanto, ao se analisar cada localização isoladamente, tais diferenças não se mantêm. Por outro lado, quando se faz ajuste considerando o volume do átrio esquerdo, os resultados permanecem os mesmos. Esses últimos dados sugerem que há alteração significativa na densidade de nervos em pacientes com FA permanente, ainda que levando em conta o aumento do átrio esquerdo.

Diversos artigos ^12 , 14 , 19 , 20^ relataram inervação autonômica aumentada em áreas eletrofisiologicamente relacionadas à FA, tais como as veias pulmonares, os seios coronários e a veia de Marshall. Esses estudos compararam apenas a densidade de nervos (simpáticos ou parassimpáticos) nessas regiões, ou em outras, com o plexo ganglionar no miocárdio atrial. No entanto, esses territórios próximos aos plexos ganglionares têm grande densidade de nervos, mas que não obrigatoriamente estão vinculados à FA. Nossos dados revelam grande concentração do SNACI nessas áreas, especialmente inervação simpática. A densidade aumentada de nervos simpáticos pode ser um potencial desencadeante de arritmia causada pela inervação próxima ao plexo ganglionar e à resultante ativação do sistema nervoso autônomo, como já demonstrado em estudos experimentais. ^12 - 20^

### Receptores muscarínicos na fibrilação atrial permanente

A estimulação dos neurônios parassimpáticos pós-ganglionares libera acetilcolina (mediador colinérgico), a qual atua nos receptores muscarínicos na membrana celular em órgãos-alvo (no caso do coração, na membrana dos miócitos). ^21^ Foram descritos cinco tipos de receptores muscarínicos (M1 a M5), cuja presença em seres humanos foi demonstrada por Wang et al., ^22^ em estudo descritivo de amostras de átrios direitos obtidas de 4 pacientes submetidos à cirurgia de revascularização do miocárdio. ^22^ No presente trabalho, a expressão de todos esses receptores (exceto M5) estava aumentada em corações de portadores de FA em comparação à dos controles. A mais significativamente alterada foi a do receptor M1, inclusive nas análises ajustadas, como apresentado na tabela 3 . Todas as localizações exibiam aumento significativo desse receptor, com exceção da junção da veia pulmonar superior esquerda. O aumento de M1 no miocárdio de pessoas com FA permanente pode estar diretamente relacionado com a fibrilação em si, e ajuda a explicar o aumento anteriormente descrito do tônus simpático por liberação de catecolaminas nos terminais nervosos simpáticos, com efeito estimulador induzido por estas. ^23^

Os receptores 2, 3 e 4 estavam aumentados nos pacientes com FA em apenas um local: 2 e 3 próximos ao coxim gorduroso superior esquerdo, e 4 na região da veia de Marshall. Além dos receptores M1 e M2, o M4 foi encontrado nos gânglios simpáticos e pode ser induzido por catecolaminas, de forma similar ao receptor M1. De acordo com estudo de Makino et al., a região da veia de Marshall tem grande número de fibras nervosas simpáticas e de gânglios parassimpáticos, e pode de fato ter um papel ligado à expressão aumentada desses receptores. ^14^ Assim, as áreas afetadas são de fato as mais relacionadas à FA; apenas o M1 parece ter alteração mais difusa, atingindo o átrio direito e o esquerdo.

Foram descritas alterações na expressão de receptores muscarínicos em modelos experimentais, o que pode sugerir que eles tenham uma função na fisiopatologia, e talvez no tratamento, da FA. Em modelo experimental de insuficiência cardíaca em cães, as densidades dos receptores M2 e M4 estavam reduzidas, e as dos receptores, aumentadas nos átrios com FA, em comparação com os sem FA. ^24^ Deve-se salientar que M2 e M4 inibem os canais de cálcio, e o M2 tem ações inotrópicas e cronotrópicas. ^21 , 22^ Assim, seria possível esperar que esses receptores estivessem diminuídos, e não aumentados, na FA permanente. O mesmo não se aplica aos receptores M1 e M3, de quem foram documentadas funções estimuladoras em outros órgãos. ^22^ O receptor M5 e suas ações são pouco conhecidos nos corações humanos, mas de todo modo quanto a ele não houve diferença entre os grupos.

Nossos resultados sugerem que o tecido miocárdico adjacente aos plexos ganglionares pode estar associado à expressão aumentada de receptores muscarínicos, exceto no caso do M5. A expressão aumentada do receptor muscarínico ocorreu mais frequentemente na porção do átrio esquerdo, onde se situa a veia de Marshall.

Embora não tenhamos avaliado função, algumas considerações sobre a fisiopatologia da FA permanente podem ser feitas com base em nossas observações morfológicas. Primeiramente, é necessário considerar a possibilidade de que as alterações que encontramos sejam não a causa, mas o efeito da FA, por mecanismo não esclarecido. Por outro lado, o desequilíbrio do SNACI, como demonstrado em estudos experimentais e eletrofisiológicos, pode ser causado por baixa atividade da inervação cardíaca autonômica (na qual a redução da área média dos nervos com manutenção geral da densidade de fibras poderia ter uma função, ainda que seja importante mencionar que não houve alteração na área de nervos), com aumento desproporcional da inervação simpática. É importante salientar que o aumento da expressão cardíaca de receptores muscarínicos, especialmente dos relacionados à atividade induzida por catecolaminas (M1, M2 e M4) e em regiões específicas relacionadas à FA (M1 e M3), aponta para a existência de possível desequilíbrio na atividade autonômica que poderia perpetuar essa arritmia de modo permanente em corações humanos, ao aumentar a sensibilidade a estímulos atriais causados pela acetilcolina.

### Receptores β-adrenérgicos na fibrilação atrial permanente e o uso de β-bloqueadores

Em que pese a grande importância do controle β-adrenérgico do ritmo cardíaco, nossos dados indicam que não há diferença na expressão de seus receptores ou da quinase GRK-5 com o uso de β-bloqueadores.

Não foi encontrada diferença significativa nos receptores β-adrenérgicos tipos 1 ou 2. Por outro lado, os receptores β3 e GRK-5 estavam bastante aumentados nas amostras de pacientes com FA permanente.

### Considerações metodológicas e limitações do estudo

Há relativamente poucos trabalhos que usam métodos anatomopatológicos para estudar arritmias cardíacas, principalmente porque grande parte das alterações subjacentes a elas são essencialmente eletrofisiológicas, com poucas repercussões morfológicas, e porque frequentemente requerem trabalhosos mapeamentos cardíacos. Porém, uma vez que tais desafios sejam encarados, esses métodos têm potencial de contribuir de forma significativa para o entendimento dessas doenças. Nossa abordagem no presente estudo foi a de verificar tipos e áreas das fibras nervosas autonômicas, a expressão de receptores muscarínicos e adrenérgicos e a quinase desses últimos (GRK-5) na fibrilação atrial humana.

Nossos achados demonstram que esse método é útil para identificar alterações eventualmente presentes (como nos receptores). Claramente, uma das limitações desse tipo de estudo é que a expressão morfológica das fibras nervosas e dos receptores não implica diretamente que sejam funcionais, mas pode-se inferir que mudanças em suas concentrações miocárdicas podem refletir alterações em sua atividade.

Vale reforçar a importância da escolha de controles adequados para os estudos patológicos: ainda que a FA em geral ocorra em pacientes como acompanhante de alguma doença estrutural, a maioria dos artigos anteriores utilizou corações normais como controles. ^11^ Desse modo, é impossível determinar com precisão suficiente quais achados são, de fato, ligados à arritmia. Para evitar esse viés, nossos pacientes-controle tinham as mesmas doenças que aqueles com FA, como se tivéssemos “excluído” as doenças acima e abaixo de uma linha de fração, deixando apenas a arritmia para explicar as diferenças. Além disso, usamos amostras de pacientes com pelo menos 2 anos desde o diagnóstico, para ter certeza de que qualquer alteração potencial fosse fixa.

## Conclusões

O aumento da inervação do sistema nervoso autônomo cardíaco intrínseco, assim como o remodelamento da expressão de receptores em regiões propensas a desencadear fibrilação atrial, podem ter uma função na condição de pacientes com fibrilação atrial permanente secundária à doença cardíaca estrutural.
