# Supplementary material for: A Remodelação do Sistema Nervoso Autônomo Cardíaco pode Desempenhar um Papel na Fibrilação Atrial: Um Estudo do Sistema Nervoso Autônomo e Receptores Miocárdicos
Source: Arq Bras Cardiol. 2021 Aug 4;117(5):999–1007. [Article in Portuguese] doi: 10.36660/abc.20200725 (PMC8682090; doi:10.36660/abc.20200725)
Supplement: Translation

# Translation: Cardiac Autonomic Nervous System Remodeling May Play a Role in Atrial Fibrillation: A Study of the Autonomic Nervous System and Myocardial Receptors

**Authors:** Ítalo Martins de Oliveira, Evilásio Leobino da Silva, Yasmin de Oliveira Martins, Hermano Alexandre Lima Rocha, Maurício Ibrahim Scanavacca, Paulo Sampaio Gutierrez

**Affiliations:** 1 Brasil Instituto do Coração (InCor), Hospital das Clínicas da Faculdade de Medicina da Universidade de São Paulo, São Paulo, SP - Brasil; 2 Brasil Hospital Messejana de Coração e Pulmão Dr. Carlos Alberto Studart Gomes, Fortaleza, CE - Brasil; 3 Brasil Hospital Geral de Fortaleza (HGF), Fortaleza, CE - Brasil; 4 EUA Harvard T.H. Chan School of Public Health , Boston - EUA

**Keywords:** Atrial Fibrillation/physiopathology, Autonomic Nervous System, Neurotransmitter Agents, Myocardium

## Abstract

**Background:**

The primary factors that originate and perpetuate atrial fibrillation (AF) are electrical and anatomical substrate alterations. However, the central mechanisms governing AF perpetuation have not been elucidated yet, which is reflected on the modest results of the treatment in patients with long persistent AF.

**Objective:**

To evaluate if human intrinsic cardiac autonomic nervous system (ICANS) remodeling, including nervous system fibers and muscarinic and β-adrenergic receptors, play a role in permanent AF.

**Methods:**

Heart necropsy samples from thirteen patients with heart disease and permanent AF and thirteen controls without AF were used. By using immunoperoxidase and histomorphometry quantification, we identified the following: the density of all fibers of the ICANS, sympathetic and parasympathetic fibers; and the percentage of myocardium positive for β-adrenergic receptors 1, 2 and 3; G protein-coupled receptor kinase-5 (GRK-5); and muscarinic receptors M1 to M5. The results were compared using ANOVA and nested ANOVA and were adjusted according to the left atrium volume for all variables, and β-blocker use to evaluate the expression of β-receptors and GRK-5.

**Results:**

There was an overall increase in the density of fibers of the ICANS (p=0.006), especially in atrial sympathetic nerve fibers (p=0.017). Only M1 muscarinic receptors were increased (5.87 vs 2.35, p=0.032). For adrenergic receptors, the results were positive for increased expression of β-3 (37.41 vs 34.18, p=0.039) and GRK-5 (51.16 vs 47.66; p<0.001). β-blocker use had no impact on β-receptor expression.

**Conclusion:**

Increased ICANS innervation and remodeling receptor expression in regions prone to triggering AF may play a role in permanent AF.

## Introduction

The primary factors that originate and perpetuate atrial fibrillation (AF) are electrical and anatomical substrate alterations, which involve many factors. In patients with AF without structural heart disease, ectopic foci in pulmonary veins have a well-defined role as triggers of paroxysmal AF.^1^ In most cases, however, AF is a consequence of a structural disease, such as ischemic heart disease, valvular disease and others, presenting hemodynamic and anatomical consequences, such as left atrial enlargement, which are related to arrhythmia progression.^1^

Fibrosis is also widely regarded as an independent factor related to persistent AF in structurally altered hearts.^2^ Nevertheless, this data does not fully explain arrhythmia, and myocardial fibrosis might be more closely related to the underlying heart disease, rather than persistent AF itself.^3^

Invasive electrophysiological assessment of the pulmonary veins (PVs) has demonstrated not only an effective heterogeneity if the refractory period, but also anisotropic conduction properties, both at the pulmonary veins and at the PV-left atrium ostia, which can provide a substrate for reentry.^4^ However, the central mechanisms governing AF perpetuation have not been elucidated yet, which is reflected on the modest results of treatment in patients with long persistent AF.^5^

Basic and clinical studies have suggested a significant participation of the cardiac autonomic nervous system in triggering and maintaining AF.^6 , 7^ The activation of the cardiac autonomic nervous system can cause important changes in the refractory period of the atria, including increased dispersion of refractoriness, which is a major mechanism for the development of persistent AF.^1 , 8 - 10^

Experimental studies show sympathetic hyperinnervation in dogs with AF, and increased sympathetic and parasympathetic innervation in areas related to this arrhythmia in animals with heart failure.^11^ A relationship between the intrinsic cardiac autonomic nervous system (ICANS) and AF has also been reported in humans, but the comparison was made with healthy patients.^2 , 12^ The possibility of alterations in fibers of the ICANS and receptors in human AF has therefore been poorly explored up to this point.

Thus, the aim of this study was to evaluate ICANS, including the sympathetic and parasympathetic fibers, and the atrial myocardial expression of the five types of muscarinic receptors, and of the three types of adrenergic receptors, as well as of G-protein-coupled receptor kinase-5 (GRK-5), which controls the expression of adrenergic receptors. We studied the hearts of patients with structural diseases and permanent AF and control cases that, importantly, were matched by the same diseases, but without AF.

## Methods

This study was guided by the principles of the Declaration of Helsinki and approved by the Scientific and Ethics Committee of the Heart Institute (InCor), #SDC 3043/07/118, University of São Paulo, School of Medicine, São Paulo, Brazil.

### Patients

We used the same samples from a previous study.^3^ We analyzed thirteen hearts from adult patients (older than 18 years of age) with recorded permanent AF (for at least 2 years)^1^ that underwent necropsy (performed less than 24 hours after death) in the Pathology Laboratory at this hospital. All the patients had underlying heart diseases: ischemic heart disease (4), valve disease (4), hypertensive cardiopathy (2), idiopathic dilated cardiomyopathy (2), or Chagas disease (1). To avoid confounding factors linked to the underlying diseases, hearts from thirteen other patients analyzed in the same laboratory were included as controls. The subjects were chosen by matching the heart diseases to those of the patients with permanent AF, but without any mention of atrial arrhythmia in their files. Patients who underwent any type of surgery or other procedures with the potential to modify cardiac structure were excluded, as were hearts from patients with congenital heart diseases.

### Heart samples

Four heart samples containing epicardium, myocardium, and endocardium were taken from each heart: at the posterior wall of the right atrium ( Figure 1A ); at the junction of the left superior pulmonary vein with the left atrium ( Figure 1B ); at the middle of the route of the vein of Marshall ( Figure 1C ); around the superior left fat pad ( Figure 1D ). These areas were chosen because these structures (fat pads, the vein of Marshall) have been implicated in AF. These sampling areas are commonly analyzed in other studies.^3 , 12 , 13^ The posterior wall of the right atrium was sampled to verify whether the alterations were diffuse in the atria. These locations are shown in Figure 1 .

**Figure 1:** – *Photorealistic image of the posterior view of the human heart. Four heart samples were collected from the following locations: A) posterior wall of the right atrium; B) junction of the left superior pulmonary vein and left atrium; C) middle route of the Marshall vein; D) superior left fat-pad.*

After conventional histological processing and embedding, four micrometer-thick sections of these samples were prepared to quantify autonomic innervation, adrenergic and muscarinic receptors and GRK-5 expression.

### Quantification of autonomic receptors

Strong positivity for adrenergic, muscarinic receptors, GRK-5 and total myocardial area was measured by automatic color detection in 3 microscopic fields in each slide. To avoid selection bias in choosing the fields, we analyzed those more distant from the slide tag. Additional analyses to verify the effects of β-blocker use and β-receptor expression were also performed.

### Quantification of autonomic nerve fibers, receptors and immunohistochemistry

Additionally, all samples of each heart were verified for the quantification of autonomic nerve fibers. The S-100 protein stains all nerves, whereas tyrosine hydroxylase (TH) only stains postganglionic adrenergic (sympathetic) fibers. Thus, like other authors,^14^ we evaluated cost-effectiveness and considered the TH positive nerves as sympathetic nerves; and parasympathetic nerves were considered to be S-100 positive and TH negative. The area of the section, as well as the area and number of nerves positive for the antibody were quantified in each slide. The following variables were then calculated: mean percent positive area (positive area/section area); mean density of positive nerves (number of positive nerves/section area); and mean area of the nerves (positive area/number of nerves). We also calculated the total number of nerve fibers (S-100 positive); sympathetic nerve fibers (TH positive); and parasympathetic nerve fibers (S-100 positive and TH negative, difference between total and sympathetic nerve fibers).

To increase the contrast between weak and strong positivity, the dilutions for the receptors and GRK-5 were supraoptimal^15^ when compared to those established in control tissues. As a control for the reactions, the primary antibody was omitted in 5 slides chosen at random. The sections were examined on an *Axiovision* 4.6 image analysis system, coupled to an *Axion imager* A1 *microscope* (both from *Carl Zeiss* , Germany), by an observer blinded to the group to which the slides belonged.

Antibody specification and dilution: muscarinic receptor 1 (AB5164) - 1:100; muscarinic receptor 2 (AB9452) - 1:800; muscarinic receptor 3 (AB9451) - 1:200; muscarinic receptor 4 (AB9219) - 1:400; muscarinic receptor 5 (AB9453) - 1:400; adrenergic receptor β1 (SC568) – 1:200; adrenergic receptor β2 (SC570) - 1:50; adrenergic receptor β3 (SC1473) - 1:20; receptor kinase GRK5 (SC 565) – 1:200; S-100 (Z0311) - 1:300; tyrosine hydroxylase (MAB318) - 1:50.

The antibody for S-100 was from *Dako* , Denmark. The antibodies for tyrosine hydroxylase and muscarinic receptors were from *Chemicon* , USA. The antibodies for the adrenergic receptors and GRK-5 were from *Santa Cruz* Biotechnology, USA.

### Statistical analysis

Initially, absolute and relative frequencies were calculated for categorical variables, and measures of central tendency and dispersion for numeric ones. Chi square and Student’s t test were used to compare cases and controls. Parametric tests were used after the Kolmogorov-Smirnov normality test was performed for all variables, and therefore robust error estimators were used in regressive models. An ANOVA One-Way was performed considering each set of equal samples to identify differences between them. Analysis of covariance was also performed for atrial dimensions and β-blocker use adjustment, when appropriate, while analyzing the individual sections. General linear models, also known as nested ANOVA, of all histological samples for each one of the individual participants, were applied to identify the impact of the principal determinant (namely, treatment, a between-subjects factor) on the various dependent variables. Finally, multiple general linear nested models were applied to all histological sections for each case. We considered significant p values to be lower than or equal to 0.05. In all models, Bonferroni adjustment in p values was performed. Analyses were done using SPSS v.23, IBM, Inc.

As in our previous study of fibrosis and histological features, since left atrial volume differs between patients with and without permanent AF, we performed a sensibility analysis with adjusted means considering the differences in left atrium size. Then, we predicted the results of each variable in hearts of any group with a given left atrium size to verify if potential differences between groups could be linked to this covariable. Additionally, β-blocker use was included for β-receptor and GRK-5 expression adjusted analysis.

## Results

The clinical, morphological, and echocardiographic characteristics of patients with permanent AF and their controls are shown in Table 1 .

**Table 1:** – Clinical and echocardiographic data from patients with permanent AF and control cases

Variables	Cases with pAF (n=13)	Controls (n=13)	p
Male patients [n/(%)]	5 (38.5)	8 (61.5)	0.24^Ϯ^
Age (years) [mean/(sd)]	67.5 (15.4)	65.5 (11.4)	0.71^¥^
**Underlying heart disease [n/(%)]**			
Ischemic heart disease	4 (30.8)	4 (30.8)	
Valve disease, including RHD	4 (30.8)	4 (30.8)	
Hypertensive cardiopathy	2 (15.4)	2 (15.4)	
Idiopathic dilated cardiomyopathy	2 (15.4)	2 (15.4)	
Chagas’ disease	1 (7.7)	1 (7.7)	
Weight (kg) [mean/(sd)]	66.5 (14.1)	63.8 (15.0)	0.67^¥^
Height (cm) [mean/(sd)]	162.4 (14.7)	160.8 (8.8)	0.78^¥^
BMI (kg/m^2^) [mean/(sd)]	25.0 (2.9)	24.5 (4.2)	0.74^¥^
Diabetes *mellitus* [n/(%)]*	3 (23.1)	3 (25.0) (n=12)	0.99^Ϯ^
Beta-blocker use	5 (38.4)	5 (38.4)	
Systemic arterial hypertension – [n/(%)]*	9 (69.2)	4 (33.3) (n=12)	0.07^Ϯ^
Left atrium volume at echo (mL) [mean/(sd)]	83.2 (38.4)	47.9 (40.8)	0.03^¥^
LV septum thickness (mm) [mean/(sd)]	10.3 (2.4)	10.4 (1.6)	0.94^¥^
LV ejection fraction [mean/(sd)]	49.8 (20.1)	46.1 (19.8)	0.67^¥^
Collagen/collagen+myocardium ratio [mean +(sd)]	0.26 (0.09)	0.23 (0.06)	0.35^¥^

pAF: permanent atrial fibrillation; n: number of cases; sd: standard deviation; RHD: rheumatic heart disease; BMI: body mass index; * no information regarding one control patient; echo: echocardiogram; LV: left ventricle. ¥ t test; Ϯ chi-square. Adapted from Oliveira IM et al.^3^

Data concerning nerve fibers and considering each sample and all samples are presented in Table 2 . When considering each location separately, we observed no difference regarding the density of intrinsic autonomic nerve fibers. The analysis considering all samples showed an increase in sympathetic nerves in patients with AF (8.53±20.25/cm^2^vs 2.67±4.57/cm^2^and p=0.04). After adjusting for the size of the left atrium, both parasympathetic nerves and the total amount of nerve fibers were also increased. Figure 2 (A and B) shows the immunoexpression of nerve fibers in our samples.

**Table 2:** – Autonomic nerve fibers from hearts of patients with permanent AF and control cases

Fibers	All (S100) (units/cm^2^)		Sympathetic nerve (TH+) (units/cm^2^)		Parasympathetic nerve (TH-) (units/cm^2^)	
Group	pAF	Control	pAF	Control	pAF	Control
RA - posterior wall	8.85±9.40	9.10±5.15	0.37±0.99	0.50±1.14	8.48±9.57	8.59±5.07
	p 0.935,	0.710^¥^	p 0.753,	0.905^¥^	p 0.971,	0.700^¥^
LA - junction of the left superior pulmonary vein	41.61±35.79	25.78±20.90	19.74±34.26	4.95±6.78	21.86±14.78	20.83±20.47
	p 0.181,	0.256^¥^	p 0.140,	0.158^¥^	p 0.884,	0.918^¥^
LA - middle of the route of the vein of Marshall	40.15±60.28	14.90±9.48	5.58±9.56	2.39±4.76	34.56±58.07	12.51±9.48
	p 0.149,	0.390^¥^	p 0.292,	0.230^¥^	p 0.189,	0.500^¥^
FP - superior left	38.05±55.72	19.25±11.95	8.42±16.07	2.85±2.82	29.62±40.56	17.47±10.53
	p 0.246,	0.637^¥^	p 0.248,	0.666^¥^	p 0.325,	0.681^¥^
Overall samples	32.16±45.76	17.26±14.20	8.53±20.25	2.67±4.57	23.63±36.77	14.80±13.27
	p 0.136^&^,	0.001^Ϯ^	p 0.044^&^,	0.017^Ϯ^	p 0.237^&^,	0.001^Ϯ^

*Data presented as mean±standard deviation. Overall locations include all samples from each heart. pAF: permanent atrial fibrillation; LA: left atrium; RA: right atrium; FP: fat pad. p value ANOVA not adjusted, ¥ ANOVA adjusted by left atrium volume; & Nested ANOVA not adjusted; Ϯ Nested ANOVA adjusted by left atrium volume.*

**Figure 2:** – *A) Nerve fibers strongly positive for tyrosine hydroxylase, thus considered to be sympathetic fibers; B) Photomicrograph of the image analysis system display showing nerves stained by S-100 protein; C and D) Negative (C) and positive (D) areas of myocardial sections with immunohistochemical reaction for muscarinic receptor 1.*

Results regarding the expression of muscarinic and adrenergic receptors and GRK-5 are presented in Table 3 . The results are divided by myocardial area for each location and atrial sample.

**Table 3:** – Muscarinic and β-adrenergic receptor expression in hearts of patients with permanent AF and control cases

Receptor	Group	RA - posterior wall			LA - midpoint of Marshall vein			LA - junction of the left superior pulmonary vein			LA - near the left superior fat pad			Overall samples		
			p	p*		p	p*		p	p*		p	p*		p	p**
M1	pAF	6.47±3.39	0.001	0.002	5.56±4.64	0.021	0.131	6.32±5.33	0.286	0.270	5.15±4.89	0.038	0.220	5.87±4.52	<0.001	0.032
	Control	2.77±1.38			2.22±1.48			4.30±4.03			2.12±0.93			2.85±2.40		
M2	pAF	7.60±5.95	0.762	0.982	5.64±3.54	0.110	0.066	7.84±4.13	0.198	0.107	5.65±2.41	0.039	0.038	6.69±4.26	0.760	0.666
	Control	6.93±5.15			3.73±2.12			14.24±16.88			3.62±2.41			7.14±9.73		
M3	pAF	43.50±19.08	0.105	0.315	37.61±20.97	0.296	0.281	41.90±18.88	0.546	0.281	31.00±13.27	0.025	0.151	38.51±18.34	0.069	0.291
	Control	31.04±18.61			29.10±18.80			46.50±19.61			20.10±9.58			31.71±19.21		
M4	pAF	9.14±5.47	0.201	0.169	9.90±6.67	0.023	0.049	7.64±4.00	0.690	0.618	8.18±11.72	0.192	0.678	8.71±7.37	0.016	0.213
	Control	5.76±4.74			4.44±4.56			8.42±5.66			3.76±1.95			5.59±5.45		
M5	pAF	18.94±11.93	0.302	0.704	12.90±11.34	0.368	0.645	20.92±22.81	0.737	0.946	12.06±9.32	0.570	0.977	16.21±14.88	0.212	0.507
	Control	14.51±9.37			8.67±12.14			18.30±15.91			9.83±10.39			12.83±12.47		
β1	pAF	43.90±12.39	0.975	0.742	47.59±21.40	0.036	<0.001	37.48±21.90	0.438	0.288	40.23±22.37	0.552	0.214	42.05±19.75	0.295	0.520
	Control	44.10±17.81			28.98±19.34			43.60±17.42			34.89±22.83			37.89±19.93		
β2	pAF	23.81±11.96	0.785	0.445	32.42±19.20	0.180	0.589	20.57±13.48	0.323	0.257	23.47±16.69	0.037	<0.001	24.80±15.61	0.408	0.081
	Control	25.48±17.51			23.04±13.88			27.63±21.32			12.38±7.00			22.14±16.46		
β3	pAF	39.32±20.29	0.911	0.469	36.36±26.36	0.422	0.940	36.45±11.81	0.351	0.281	37.50±18.53	0.177	0.314	37.41±18.17	0.406	0.039
	Control	38.40±20.45			29.04±20.40			42.38±19.10			26.89±20.31			34.18±20.53		
GRK5	pAF	49.81±18.49	0.899	0.976	44.84±18.78	0.999	0.147	53.43±15.28	0.796	0.862	55.45±16.53	0.086	0.320	51.16±17.17	0.284	<0.001
	Control	50.53±7.61			44.85±19.75			52.95±13.26			43.29±18.00			47.66±15.39		

Data presented as the mean proportion (%) ± standard deviation. pAF: permanent atrial fibrillation; LA; left atrium; RA: right atrium. p value ANOVA not adjusted. *Anova adjusted by left atrium volume for M1 to M5, and by left atrium volume and β-blocker use in β1 to β3 and GRK5.**Nested Anova adjusted by left atrium volume for M1 to M5 and by left atrium volume and β-blocker use in β1 to β3 and GRK-5.

Immunostaining for muscarinic receptors is shown in Figure 2- C and D . There was no remarkable difference between the subepicardial and subendocardial regions. In hearts from patients with permanent AF, the expression of all types of muscarinic receptors (except type 5) was increased in at least one location. We observed more changes in the left superior fat pad and the oblique vein of the left atrium (vein of Marshall). Nevertheless, after adjusting for left atrial size, only the difference in M1 expression in the right atrium (and, consequently, the overall evaluation) and M2 near the fat pad remained significant.

Concerning β-adrenergic receptors and GRK-5, no difference was found in the overall analysis of the β-adrenergic subtypes 1 and 2 (only an increase in one sample each). However, β-3 and GRK-5 presented increased expression in all samples in the adjusted analysis. No difference was detected between patients who were taking β-blockers and those who were not (data not shown).

## Discussion

### The fibers of the ICANS in permanent AF

The ICANS is a neural network composed of nerve fibers and ganglia plexuses (GP) (sympathetic and parasympathetic) found in the heart and large adjacent veins.^16^ ICANS plays an important role in the physiopathology of AF, as demonstrated by electrical stimulation or parasympathomimetic injections.^17^ The current data reflects not only the activation of either a sympathetic or parasympathetic pathway, but also a change in the balance between their actions that is involved in the initiation of AF.^8 , 18^

In this study, we performed a comprehensive analysis of ICANS, focusing both on the nerves and on the muscarinic and beta-adrenergic receptors. We observed an increase in the amount of autonomic nerve fibers, especially atrial sympathetic nerves. However, when analyzing each location in isolation, these differences were not maintained. Moreover, when we adjusted for left atrium (LA) volume, the results remained the same. These last results also indicate that there is a significant change in nerve density in patients with permanent AF, even taking LA enlargement into consideration.

Several articles^12 , 14 , 19 , 20^ have reported increased autonomic innervation in areas related to AF in terms of electrophysiology, such as the pulmonary veins, the coronary sinus, and the vein of Marshall. These studies only compared the nerve density (parasympathetic or sympathetic) in these regions, or in other areas, with the GP in the atrial myocardium. However, these regions, close to GP, are described as having higher nerve density, but they are not necessarily related to AF. Our results reveal a higher concentration of ICANS at these regions, especially sympathetic innervation. The greater sympathetic density of nerves may be a potential trigger of arrhythmia caused by innervation close to the GP, and the resulting activation of the autonomic nervous system that has already been demonstrated in experimental studies.^12 - 20^

### Muscarinic receptors in permanent atrial fibrillation

The stimulation of the postganglionic parasympathetic neurons releases acetylcholine (cholinergic mediator), which acts on muscarinic receptors in the cell membrane in target organs (in the heart´s case, these would be the myocytes). Five types have been described.^21^ The presence of all of these receptor types (M1 to M5) in the human heart was demonstrated by Wang et al.^22^ in a descriptive study of right atrial samples from 4 patients undergoing coronary artery bypass surgery.^22^ In the present study, the expression of all receptors (except M5) was increased in the hearts of patients with AF compared to the expression in the hearts of the controls. The expression of the M1 receptor was the most significantly altered, even in adjusted analyses, as shown in Table 3 . All locations exhibited significant increase of this receptor, except at the junction of the left superior pulmonary vein. The increase in M1 in the myocardium of patients with permanent AF can be directly related to the permanent AF itself, which helps to explain the previously described increase in sympathetic tonus by the release of catecholamine in the sympathetic nerve endings, with a catecholamine-induced stimulatory effect.^23^

Receptor types 2, 3, and 4 were increased in patients with AF in only one location: 2 and 3 were increased near the superior left fat pad, and 4 was increased in the region of the vein of Marshall. In addition to the M1 and M2 receptors, M4 receptors have been found in the sympathetic ganglia and may be catecholamine-induced, similar to the M1 receptor. According to the study by Makino et al., the vein of Marshall has increased sympathetic nerve fibers and parasympathetic ganglia, and it may have an actual role linked to the enhanced expression of these receptors.^14^ Thus, these affected areas are the ones that are actually more related to AF; only M1 seems to have a more diffuse alteration, reaching both the right and the left atria.

Changes in the expression of muscarinic receptors have been described in experimental models, which may suggest its role in the physiopathology, and perhaps int he treatment of AF. In an experimental study of canine heart failure models, the densities of the M2 and M4 receptors were reduced in atria with AF, and M3 receptors were increased compared to those in samples without AF.^24^ It is noteworthy that M2 and M4 inhibit calcium channels, and M2 has inotropic and chronotropic actions.^21 , 22^ Thus, one would expect for these receptors to be decreased, and not increased, in permanent AF. The same does not apply to M1 and M3 receptors, which have been documented in other organs as having stimulatory functions.^22^ The M5 receptor and its action in the human heart are poorly understood, but the M5 receptor did not differ between the groups.

Our results suggest that the atrial myocardial tissue underlying a GP may be associated with increased muscarinic receptor expression, except in the case of M5. Increased muscarinic receptor expression occurred more often in the portion of the left atrium related to the vein of Marshall.

Despite the fact that we did not evaluate function, some considerations about the physiopathology of permanent AF in humans can be made based on our morphological observations. First, it is necessary to consider the possibility that the changes we found may not be the cause, but rather the effect of AF, by an unclear mechanism. In contrast, the imbalance of the ICANS, as demonstrated in experimental and electrophysiological studies, can be caused by lower activity of cardiac autonomic innervation (in which the reduction of the mean nerve area and the maintenance of the overall density of fibers could have an influence, although it must be mentioned that there was no alteration in the nerve area) with a disproportionate increase in sympathetic innervation. More importantly, increased myocardial expression of muscarinic receptors, especially those related to catecholamine-induced activity (M1, M2, and M4), and in specific regions related to AF (M1 and M3), indicates a possible imbalance in autonomic activity, which could perpetuate this arrhythmia in a permanent manner in human hearts by increasing the sensitivity to atrial stimulus caused by acetylcholine.

### β-adrenergic receptors in permanent atrial fibrillation and the use of β-blockers

Despite the great importance of β-adrenergic control of heart rhythm, our data indicate there was no difference in the expression of these receptors or their kinase GRK-5 with the use of β-blockers.

No important differences were found in β-adrenergic types 1 or 2. However, the β3 receptors and GRK-5 kinase were strongly increased in the samples with permanent AF.

### Methodological considerations and study limitations

Relatively few studies use pathological methods to study cardiac arrhythmias, mainly because most of the changes that underlie them are essentially electrophysiological, with few morphological repercussions, and because they frequently require laborious cardiac mapping. Once these challenges are faced, however, such methods have the potential to bring significant contributions to the understanding of these diseases. Our approach in this study was to verify types and areas of the autonomic nerve fibers, the expression of muscarinic and adrenergic receptors, and the kinase for adrenergic receptors (GRK-5) in human AF.

Our findings demonstrate that this method is useful to identify alterations when they are present (such as those observed with receptors). Clearly, one of the limitations of this kind of study is that the morphological expression of nerve fibers and receptors does not directly imply they are functional, but we can infer that changes in their myocardial concentration may reflect changes in their activity.

It is worth reinforcing the importance of choosing adequate controls for pathological studies: although AF usually occurs in patients with an underlying disease, most previous reports have used normal hearts as controls.^11^ Thus, it is not possible to determine with enough precision which findings are actually related to the arrhythmia. To avoid such bias, our control patients had the same diseases as the patients with AF, as if we had “excluded” the disease both above and below the line in a fraction, leaving only the arrhythmia as an explanation for the differences. Additionally, we utilized samples from patients with permanent AF, with at least 2 years since the time of diagnosis, aiming to be certain that any potential alterations were established.

## Conclusions

Increased ICANS innervation and receptors expression remodeling in regions prone to trigger AF may play a role in the condition of patients with permanent AF, secondary to structural heart disease.
